# USING FUNCTIONAL BRAIN IMAGING DATA TO PREDICT FALLS IN OLDER ADULTS WITH MULTIPLE SCLEROSIS

**DOI:** 10.1093/geroni/igae098.0147

**Published:** 2024-12-31

**Authors:** Roee Holtzer

**Affiliations:** Yeshiva University and Albert Einstein College of Medicine, Bronx, New York, United States

## Abstract

Background: Falls are common and debilitating among older adults with multiple sclerosis. However, literature concerning modifiable risk factors of falls in this population is scarce. The current study was designed to address this gap in knowledge. We examined whether brain hemodynamic responses during dual-task-walking predict falls during longitudinal follow-up in older adults with multiple sclerosis with relapsing-remitting and progressive subtypes. Methods: Participants with relapsing-remitting (n=53, mean age=65.02±4.17ys, %female=75.5) and progressive (n=28, mean age=64.64±4.31ys, %female=50) multiple sclerosis subtypes completed a dual-task-walking paradigm. In addition, participants reported falls during longitudinal follow-up using a well-validated structured monthly telephone interview. Functional-near-infrared-spectroscopy (fNIRS) was utilized to assess oxygenated hemoglobin (HbO) in the prefrontal cortex during active walking under single and dual-task conditions. Results: General Estimating Equations models, adjusted for confounders, indicated that higher HbO under dual-task-walking was significantly associated with a reduction in the odds of reporting falls among participants with relapsing-remitting (OR=.472, p=.004, 95%CI=.284 to.785), but not progressive (OR=1.056, p=.792, 95%CI=.703 to 1.588) MS. In contrast, faster stride velocity under dual-task-walking was significantly associated with a reduction in the odds of reporting falls among progressive (OR=.658, p=.004, 95%CI=.495 to.874), but not relapsing-remitting (OR=.998, p =.995, 95%CI=.523 to 1.905) MS. Conclusion: Compensatory reallocation of brain resources under dual-task walking may provide protection against falls for older adults with multiple sclerosis, relapsing-remitting subtype. These findings have important implications for fall risk assessment and intervention procedures in older adults with multiple sclerosis.

